# Next‐generation agents for fluorescence‐guided glioblastoma surgery

**DOI:** 10.1002/btm2.10608

**Published:** 2023-10-11

**Authors:** Cristina Chirizzi, Serena Pellegatta, Alessandro Gori, Jacopo Falco, Emanuele Rubiu, Francesco Acerbi, Francesca Baldelli Bombelli

**Affiliations:** ^1^ Department of Chemistry, Materials and Chemical Engineering “Giulio Natta” Politecnico di Milano Milano Italy; ^2^ Unit of Immunotherapy of Brain Tumors Fondazione IRCCS Istituto Neurologico Carlo Besta Milan Italy; ^3^ Unit of Neuroncology Fondazione IRCCS Istituto Neurologico Carlo Besta Milan Italy; ^4^ National Research Council of Italy, Istituto di Scienze e Tecnologie Chimiche (SCITEC‐CNR) Milan Italy; ^5^ Neurosurgical Unit 2, Department of Neurosurgery Fondazione IRCCS Istituto Neurologico Carlo Besta Milan Italy; ^6^ Experimental Microsurgical Laboratory, Department of Neurosurgery Fondazione IRCCS Istituto Neurologico Carlo Besta Milano Italy

**Keywords:** fluorescent‐guided surgery, glioblastoma, neurosurgery, novel fluorescent probes, patients' survival

## Abstract

Glioblastoma is a fast‐growing and aggressive form of brain cancer. Even with maximal treatment, patients show a low median survival and are often subjected to a high recurrence incidence. The currently available treatments require multimodal management, including maximal safe surgical resection, followed by radiation and chemotherapy. Because of the infiltrative glioblastoma nature, intraoperative differentiation of cancer tissue from normal brain parenchyma is very challenging, and this accounts for the low rate of complete tumor resection. For these reasons, clinicians have increasingly used various intraoperative adjuncts to improve surgical results, such as fluorescent agents. However, most of the existing fluorophores show several limitations such as poor selectivity, photostability, photosensitization and high costs. This could limit their application to successfully improve glioblastoma resection. In the present perspective, we highlight the possibility to develop next‐generation fluorescent tools able to more selectively label cancer cells during surgical resection.

**Translational Impact Statement**

The present study explores recent advancements in the design of innovative fluorescent tools, with the aim of significantly improving the intraoperative visualization of tumors during glioblastoma resection. These next‐generation molecules have the potential to reduce residual tumors in glioblastoma patients, ultimately leading to a decrease in tumor recurrence rates and patient mortality.

## 1 INTRODUCTION

**FIGURE 1:**
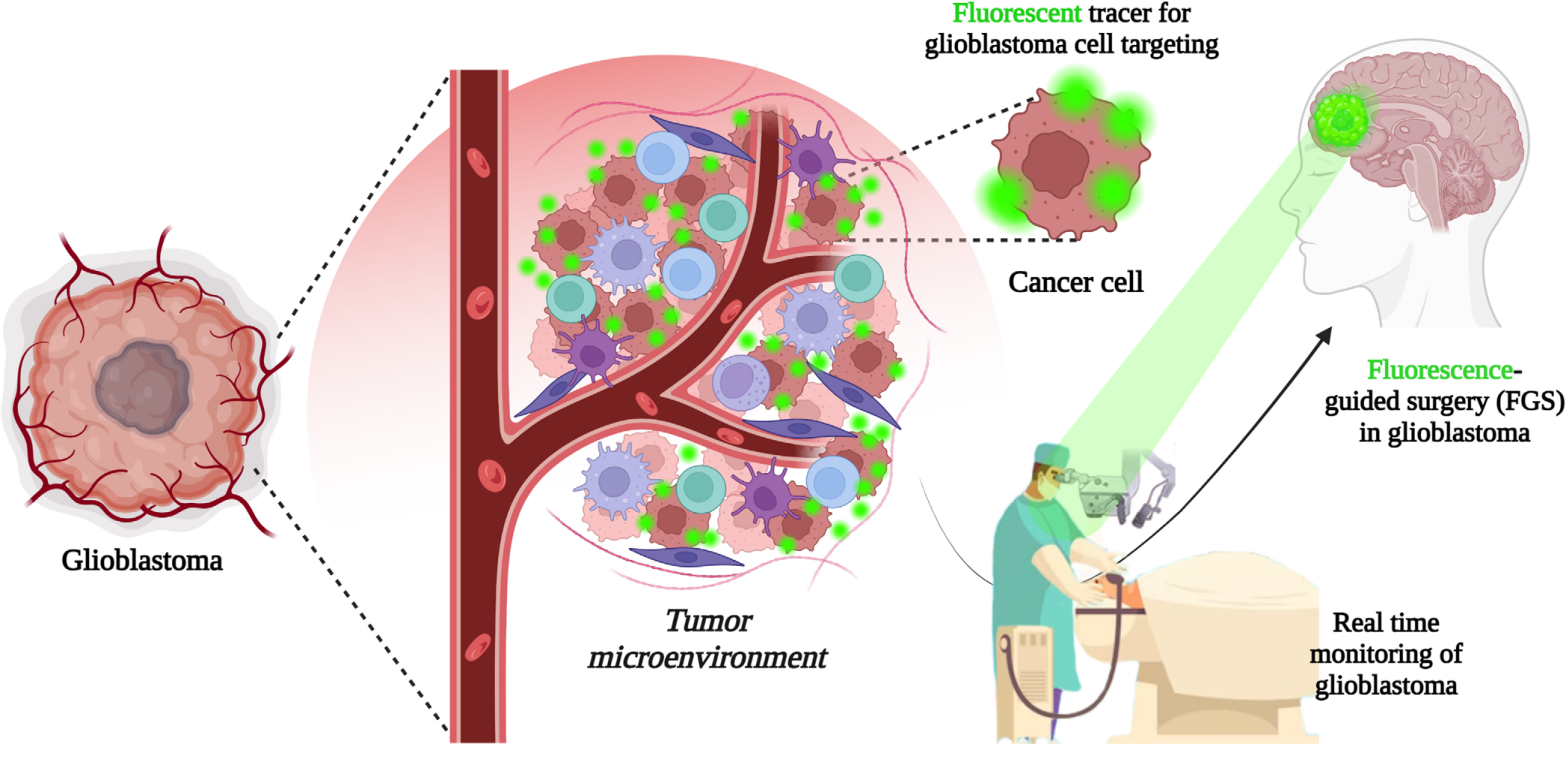
Schematic illustration of glioblastoma microenvironment and development of ideal fluorescent probes for fluorescence‐guided surgery. Created with BioRender.com.

The new classification (WHO 2021) of central nervous system (CNS) tumors has required the introduction of molecular approaches to define new subtypes and confirm rare tumors. Astrocytic tumors are grouped according to IDH mutations and include astrocytoma (IDH‐mutant) and glioblastomas (IDH‐wild type).^1^ Glioblastoma is the highest and most common form of malignancy affecting the CNS, and it represents one of the deadliest forms of adult cancer. Indeed, even with maximal treatment, defined as maximal safe surgical resection followed by radiotherapy and adjuvant temozolomide (TMZ),^2^ patients show a low median survival rate corresponding to a dismal 2‐year survival of 26%–33%.^3^ In addition, they are often subjected to a high recurrence incidence.^3^ In fact, the exclusive molecular characteristics of this tumor make the current standard of treatment of care ineffective, and recurrence is almost inevitable. The complex intratumoral heterogeneity at the genetic, biological, and functional levels, together with the tumor microenvironment, one of the most critical regulators of immune escape, is a crucial factor in making glioblastoma extremely resistant to treatments, including immunotherapy. This complex scenario is also combined with the concomitant changes induced in the microenvironment such as modification of the stromal architecture, active degradation of normal brain matrix, and immune modulation to bypass surveillance, detection, and destruction.^4^ In addition, glioblastoma cells show a bursting tendency to infiltrate into the surrounding normal brain tissues of the tumor.^5^ For all these reasons, the identification of tumor margins is very hard to achieve and particularly challenging for neurosurgeons. Even if the surgical resection is not curative, it is one of the most important factors significantly associated with overall survival (OS) and progression‐free survival (PFS) among glioblastoma patients.^6^ In this context, the crucial aim of the surgery is the combination of successful removal and preservation of the brain function, as more than 50% of glioblastoma tumors are placed near eloquent cerebral structures.^7^ Recent studies demonstrated that an extent of resection (EOR) of 78% is enough to guarantee an improved OS,^8^ but only the aggressive EOR of >98% results in a further enhanced median OS ranging from 52 to 86 weeks in newly diagnosed glioblastoma.^9^ However, the ambiguous delimitation of tumor margins is again the cause for difficult and rarely occurring complete tumor resection.^10^ Unfortunately, current technological tools such as neuronavigation,^11^ intraoperative brain MRI,^12^ and ultrasound^13^ seem to be not enough to improve intraoperative guidance and are often associated with important limitations.^14^ Thus, technical improvements for enhancing intraoperative tumor identification are strongly required. Recently, optical fluorescence imaging has emerged as a cost‐effective and time‐efficient method able to accurately guide brain surgery with a consequent EOR maximization^15^, ^16^ (Figure 1).

The first application of fluorescence‐guided surgery (FGS) for glioblastoma goes back to 1947 when Moore observed a higher concentration of fluorescein in malignant glioma tissues using a wood lamp.^17^ This approach was modernized by Walter Stummer some decades later by describing the use of 5‐aminolevulinic acid (5‐ALA) for FGS.^18^ This opened doors for its approval as an oral intraoperative agent for fluorescence‐guided neurosurgery.^19^ The translation of fluorescence in brain surgery has been exponentially growing. Currently, a limited number of fluorophores are being employed in clinical practice, including mainly 5‐ALA, sodium fluorescein (SF),^15^ and, more recently, indocyanine green (ICG).^20^ These tracers have distinct mechanisms of action. In detail, 5‐ALA and SF are characterized by intracellular uptake and extracellular accumulation,^21^, ^22^ respectively, while ICG can exhibit both pathways as widely reported.^20^, ^23^ To date, several other dyes are undergoing clinical trials and are under investigation (see Section 2.2). However, despite positive outcomes provided by these agents currently used for FGS, there is still ongoing research focused on the development of novel fluorophores to potentially overcome the limitations of current tracers. In particular, researchers are prompted to investigate innovative probes able to more specifically accumulate into the tumor region, with a greater fluorescence efficiency, deep tissue penetration, adequate aqueous stability and plasma protein binding, and, importantly, high biocompatibility.

In the current perspective, we first discuss the applicability, benefits, and mechanisms of established fluorescent agents. Based on the limitations of current FGS, we suggest the exploration of next‐generation tracers, which are clinically translatable and show ideal properties to improve glioblastoma surgery. To enhance fluorescence properties, cancer selectivity, and uptake, the proposed agents exploit different platforms, including peptide‐based compounds,^24^, ^25^, ^26^ lipid antitumor agents (alkylphosphocholine [APC] analogs),^27^, ^28^ inorganic materials (lanthanide metal complexes),^29^ and nanosystems (RGD peptide‐decorated nanocarriers, theranostic photonic nanoprobes, polymer‐based nanoparticles [NPs], etc.).^30^, ^31^, ^32^

## 2 ESTABLISHED FLUORESCENT AGENTS FOR FGS

### 2.1 Description and limitations of clinically available tracers, clinical data

In 1982, Murray et al., with a series of 23 brain tumor cases, set the stage for fluorescence‐guided neurosurgery by reporting a sensitivity of 84.7% for SF in identifying cancerous tissues.^33^ If Murray had thrown down the gauntlet, 24 years later, Stummer et al. seized it with a Phase III multicenter randomized clinical trial on the impact of 5‐ALA in high‐grade glioma (HGG) surgery.^34^ Their multivariate analysis demonstrated that complete resection of the enhancing portion of HGG occurred in 65% of patients assigned to the 5‐ALA group versus 36% in those assigned to conventional white light. Furthermore, the authors observed a significantly higher 6‐month PFS in patients undergoing 5‐ALA‐assisted resection.^34^

5‐ALA is an FDA‐approved intraoperative fluorescent dye.^35^ Specifically, it is a natural precursor of hemoglobin and elicits the synthesis of porphyrins, such as protoporphyrin IX (PpIX), a fluorescent substance capable of accumulating within glioma tissues.^16^ 5‐ALA tumor tissue buildup occurs for two main reasons: first, the enhanced porphyrins' uptake and metabolism by tumor cells and, second, the disruption of blood–brain barrier (BBB).^10^ 5‐ALA accumulation may also be explained by the typical lower ferrochelatase activity of tumor cells.^36^ Intraoperative 5‐ALA‐dependent fluorescence varies from a dark red color, at the tumor center, to a faint pink, at the tumor margins, whereas the malignant tissue becomes less dense, the dye's accumulation starts to abate. Because of its intrinsic mechanism of action, 5‐ALA is the most specific intraoperative dye for HGG; nevertheless, its applications to other malignant brain tumors are really limited because of its unpredictability of fluorescence enhancement, which is independent of cancer cellularity and aggressivity. 5‐ALA requires a surgical microscope connected to a xenon light source that can emit from 375 to 410 nm wavelength light to excite PpIX, and a filter to visualize the tumor fluorescence with emission peaks at 635–704 nm.^16^ At present, available surgical microscopes require considerable darkening of the operative field to guarantee the best visualization of 5‐ALA fluorescence.^37^ This may compromise neurovascular structure identification, resulting in unexpected bleedings, whose management may be challenging under darkfield conditions. On the other side, the development of a new blue filter in the exoscope provided a significantly better visualization than standard operative microscopes in the identification of vessels, parenchyma, and surgical instruments for both superficial and deep surgical fields.^38^ However, it has been reported that prolonged exposure to blue light may diminish 5‐ALA fluorescence. Moreover, occurring at the surface, such a photobleaching phenomenon may be lessened by the resection of a few cell layers with consequent exposure of the underlying brightly fluorescent tissue. Numerous systematic reviews evaluated 5‐ALA's role in malignant brain tumors, largely focusing on HGGs. In this regard, as reported by Gandhi et al., 5‐ALA has proven to increase GTR rates by 26.3%.^39^ Nonetheless, 5‐ALA still possesses some intrinsic pitfalls, such as the average high cost per application, the need to administer the drug orally at a dose of 20 mg/kg body weight and at least 3 h before surgery, and the 24 h‐lasting photosensitizing effect, which limit its application; indeed, this photosensitizing effect can constitute a pitfall of this dye as it requires maintaining a dark environment for the patient in the first 24 h, limiting early mobilization in the postoperative course. Other reported adverse events are liver dysfunction and temporary hypotension.^36^

SF represents another fluorescent dye, approved by the FDA for ophthalmologic purposes and still used off‐label in most of the countries in neuro‐oncological surgery. In Italy, instead, the Italian Drug Agency (AIFA) has approved the use of SF as a fluorescent tracer for aggressive CNS tumors.^40^ SF accumulates in the extracellular space of CNS BBB‐damaged areas.^41^ Theoretically, this unspecific mechanism of action may result in reduced accuracy in terms of tumor identification. However, Murray et al. assessed the accuracy in 1982, analyzing 183 fluorescent and nonfluorescent biopsies in 23 patients, with a resulting sensitivity and specificity of 96% and 81%, respectively. SF has also been associated with an increase in the EOR, which ranged from 82.8% to 100% in the FLUOGLIO study by Acerbi et al. In fact, concerning tumor tissue identification at the margins, the authors found a sensitivity and specificity of 80.8% and 79.1%, with a positive predictive value and a negative predictive value of 80.8% and 79.1%,^42^ respectively. Similar values were obtained by several other groups.^43^ In addition, as recently reported by Smith et al. in a meta‐analysis focusing on the role of SF in HGG surgery, the increase of GTR was 29.5% in the SF group, compared with white light control groups.^44^ The development of specific visualization filters in the operative microscopes has allowed a reduction in the dose of injected fluorescein (5 mg/kg), guaranteeing to perform surgery in yellow light as this fluorescence module allows for a visualization of nonfluorescent tissue in natural color, with a correct anatomical depiction and the possibility of performing ordinary hemostasis with the filter activated. Furthermore, because of its vascular and unspecific enhancement, SF applications have incredibly widened, including all tumor histotypes characterized from a BBB damaging, as predictable with preoperative MRI, and lesions at high metabolism, as detectable from amino acid PET.^37^ Moreover, in comparison with 5‐ALA, SF displays some advantages, such as the lower cost per vial, the possibility of intravenous administration at the induction of general anesthesia, allowing its utilization even in an urgent setting, the renal excretion within 24 h, and the lack of postoperative skin photosensitivity.^40^

**FIGURE 2:**
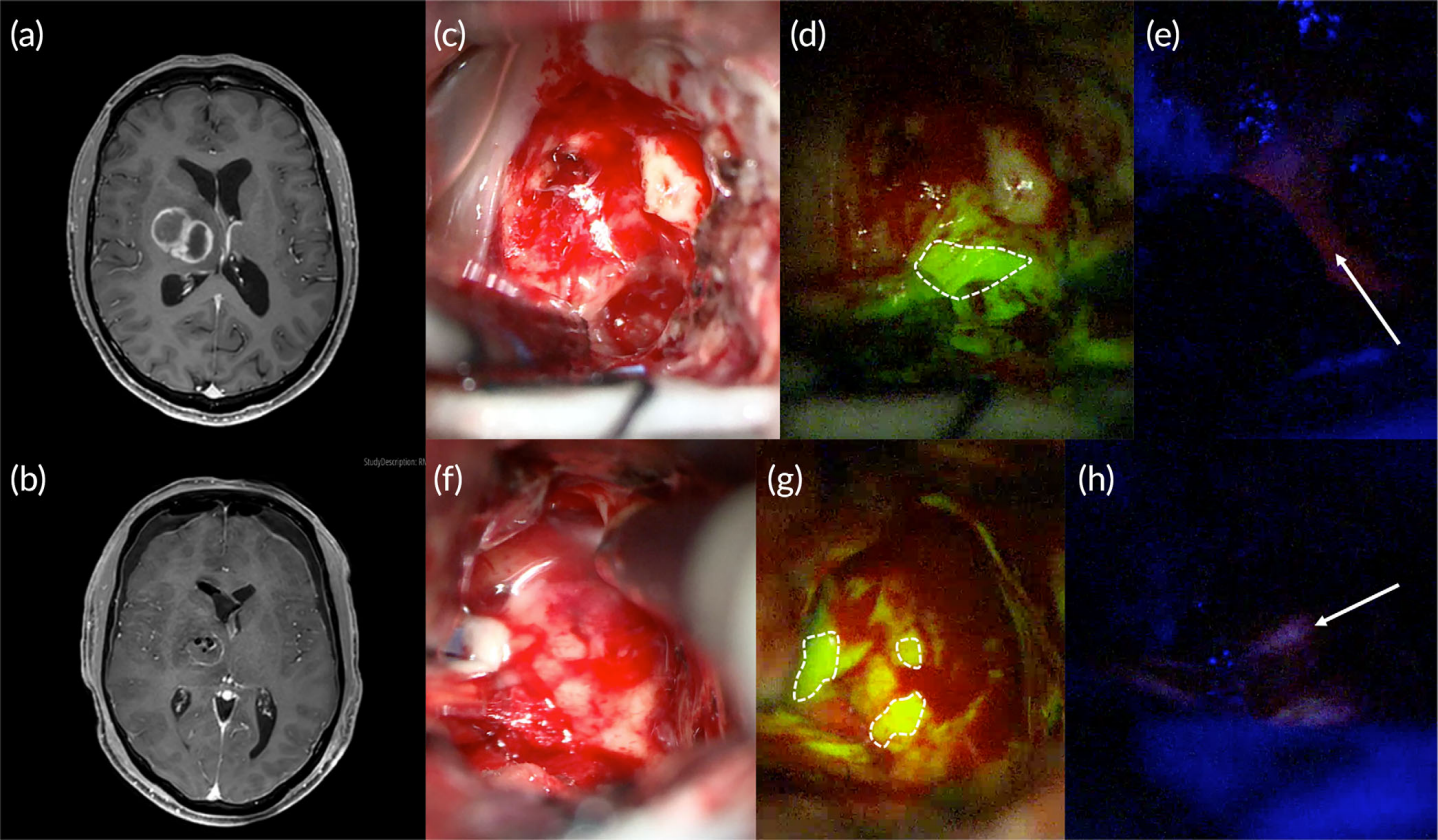
A representative case of a right thalamic glioblastoma in a 32‐year‐old male patient submitted to surgical resection under intraoperative fluorescein and 5‐ALA visualization. (a) Preoperative axial post‐contrast T_1_ MRI showed an irregular, contrast‐enhancing lesion in the right thalamus, highly suspect for high‐grade gliomas. (b) Postoperative post‐contrast T_1_ MRI showing a gross‐total resection of the contrast‐enhanced lesion. (c–h) Intraoperative pictures demonstrating the increased visualization assured by the use of fluorescein (5 mg/kg injected iv at patient intubation) and 5‐ALA (20 mg/kg, orally administered 5 h before surgery), using specific filters (Y560 or B400, Kinevo Microscope; Carl Zeiss Meditec) integrated into the surgical microscope, compared with white light illumination. Initial visualization of the center of the tumor (c–e) under Y560 and B400 filters allows us to better recognize the pathological tissue as a bright fluorescent area both with fluorescein (green‐yellow area with the dotted line in d) and with 5‐ALA (red area highlighted by an arrow in e) as compared with white light illumination (c). At the marginal area of resection (f–h), the residual pathological tissue is still better visible as a faintish fluorescent area both with a Y560 filter (yellowish area in dotted lines in g) and with a B400 filter (faint pink highlighted by an arrow in h) as compared with white light illumination (f).

Some authors have suggested that the concomitant use of 5‐ALA and SF could overcome their specific limitation, with a possible advantage in glioblastoma resection^45^ (Figure 2).

ICG is a hydrophobic cyanine near‐infrared (NIR) fluorophore, first developed in ophthalmology and approved for retinal blood vessel visualization, thanks to its ability to bind plasma proteins and to remain in the intravascular space.^46^, ^47^ In addition, ICG has been extensively used to intraoperatively identify patency and flow characteristics of intracranial blood vessels during neurovascular surgery.^48^, ^49^ ICG peaks of emission and excitation are 780–835 and 805–810 nm, respectively, and require an NIR imaging device for visualization. ICG is removed through biliary excretion, and no significant side effect has been reported.^20^ The oncological application of ICG, known as second window indocyanine green (SWIG), has been used in different extracranial neoplasms, such as lung, prostate, breast, ovarian, colorectal, pancreatic, and esophageal cancers. SWIG is the youngest dye to come onto the neuro‐oncological stage, with an initial wide application and studies in gliomas, meningiomas, pituitary adenomas, chordomas, and craniopharyngiomas.^20^ In the SWIG setting, ICG, administered at a dose of 5 mg/kg 24 h before surgery, may permeate neoplastic tissues via a mechanism similar to that of MRI contrast medium. A noteworthy advantage of the NIR fluorescence is the increase in tissue penetration, which allows for precocious tumor visualization, even before the dura opening. ICG mechanism of action is based on the enhanced permeability and retention (EPR) principle, according to which ICG accumulates in brain areas with defective BBB.^46^, ^50^ The implementation of SWIG has been reported by Cho et al. in a series of 15 glioma patients, in which SWIG demonstrated a 98% sensitivity and 45% specificity for detecting neoplastic infiltration in biopsy specimens.^20^ Another study showed that SWIG could demonstrate a sensitive detection of extra‐axial tumors as well, with strong fluorescence in 14 out of 18 cases of meningioma.^51^ Intraoperative ICG administration may also be performed to identify hypervascular tumor remnants after tumor debulking. Despite its increasing application in clinical practice, SWIG is not without flaws: the weakness of the dye's signal and the potentially limited tracer bioavailability due to its binding to plasma proteins still limits its widespread use.^52^ Furthermore, because of its protocol in application, SWIG cannot be used in urgent setting.

A comprehensive comparison between 5‐ALA, SF, and SWIG for neuro‐oncological surgery is summarized in the table below (Table 1).

**TABLE 1:** Comparison between 5‐aminolevulinic acid, sodium fluorescein, and second window ICG for neuro‐oncological surgery.

	5‐ALA	SF	SWIG
Type of substance	Drug: 5‐aminolevulinic acid (porphyrin precursor)	Dye: sodium salt of fluorescein	Dye: hydrophobic cyanine near‐infrared (NIR) fluorophore
Mechanism of action	Selectively elicits the synthesis of fluorescent porphyrin IX in tumoral cells	Passage throughout the damaged blood–brain barrier	Bind plasma proteins and remain in the intravascular space with accumulation in areas of BBB alteration
Dosage (mg/kg)	20	5	5
Time and mode of injection	3 h before surgery, oral administration	Upon completion of anesthesia procedures, iv administration. The only tracer that can be used in an urgent setting	24 h before surgery, iv administration
Fluorescence visualization	Specific filters (darkening of the operative field)	Specific filters unless very high dosage (yellow light allows for a visualization of nonfluorescent tissue in natural color, with a correct anatomical depiction)	Specific infrared filters (darkening of the operative field)
Tumor histotypes	HGG; inconstant visualization of cerebral metastases	All tumors with c.e. at preoperative imaging	Gliomas and meningiomas; potentially, tumors with high abnormal microvasculature
Pros	High specificity for malignant glioma	Favorable learning curve, the feasibility of performing surgery under yellow light, high predictability of SF enhancement	Increase of tissue penetration to detect peripheral tumor infiltration
Cons	Poorly extendable to other histotypes because of its low predictability	Risk of extravasation and reduction of specificity	Weakness of dye signal
Effect on GTR rate	65% vs. 36% (white light) (RCT)	85%–100% (prospective nonrandomized study)	No definitive data available
Effect on PFS	6 PFS 41% (RCT)	12 PFS 50% (prospective nonrandomized study)	No definitive data available
Effect on OS	Median OS 17.9 months (secondary endpoints)	Median OS 16 months (secondary endpoints)	No definitive data available
Costs	Around €900 for each vial	Around €8 for each vial	Around €800 for each vial
Contraindications	Severe liver dysfunction. To be noted the photosensitizing effect for the first 24 h	Allergy, stage IV/V of chronic kidney disease	Hepatobiliary insufficiency

Abbreviations: 5‐ALA, 5‐aminolevulinic acid; C.E, contrast enhancement; HGG, high‐grade glioma; IV, intravenous; OS, overall survival; PFS, progression‐free survival; RCT, randomized clinical trial; SF, sodium fluorescein; SWIG, second window indocyanine green.

### 2.2 Experimental tracers used in clinic

Currently, as discussed above, the main fluorophores rely on vascular anomalies, exploiting either the diseased tumoral vasculature or hypercellularity, which is another cancer hallmark.^53^

Recently, the identification of complex molecular signatures of malignant gliomas has led to the development of diverse targeted therapeutic regimens, with their respective translation into multiple clinical trials.^54^ Chemical‐, peptide‐, antibody‐, and NP‐based probes have been designed to target specific molecules in gliomas and then be visualized with multimodality molecular imaging techniques.^53^ Thus, multiple molecules of interest can now be noninvasively imaged to guide targeted therapies, with consequent potential survival benefit.^55^

Anti‐epidermal growth factor receptor (EGFR) antibody‐conjugated IR dye (IRDye800) was studied and applied in the neuro‐oncological field. This tracer has been evaluated in both in vitro and clinical models. Miller et al. presented the first Phase 1 in vivo clinical trial demonstrating the feasibility and safety of antibody‐based imaging for contrast‐enhancing glioblastomas with favorable results in terms of safety and tumor visualization.^56^

Tumor Paint BLZ‐100, a tumor ligand chlorotoxin (CTX) conjugated to ICG, has shown the potential to be a targeted contrast agent and is a candidate molecule for FGS of glioma and other tumor types.^57^ Detection cameras have been developed to highlight small concentrations of ICG molecules and enhance tumor moieties. After proof‐of‐concept and preclinical studies,^57^ clinical applications were conducted. In the first phase, in one study by Patil et al., tozuleristide (BLZ‐100) was applied in an adult population harboring a newly diagnosed or recurrent glioma.^58^ Patients received a single intravenous dose, at a different posology, of tozuleristide 3–29 h before surgery; fluorescence in situ images of tumor and cavity before and after resection and of the excised tissues, that is, ex vivo, were acquired, along with safety and pharmacokinetic (PK) measurements. This study showed that tozuleristide is well‐tolerated at doses up to 30 mg; the preliminary imaging data showed that contrast could be achieved within a few hours of dosing. Coupled with an adequately sensitive NIR imaging device that can be continually used during neurosurgery, fluorescence guidance with tozuleristide could have the potential to highlight residual cancer and increase the EOR, while minimizing damage to the normal brain.^58^ There was no drug‐related serious adverse event. In subjects who received BLZ‐100 within the optimal imaging dose range, tumors were positive for fluorescence in 18 of 23 cases (80%) on a wide range of histologic subtypes. On ex vivo analysis, the tracer's sensitivity was 82% and specificity was 89%. After evaluation of PKs, the established optimal dose for further studies was decided of being 15 mg/m^2^, iv administered 24 h before surgery, both in adult and pediatric populations. Based on preliminary encouraging results, a randomized blinded study (BB‐006) evaluating fluorescence detection of BLZ‐100 was initiated. The accrual goal is 114 patients, and enrollment is currently underway. The primary objective of the study is to collect data on the sensitivity and selectivity of BLZ‐100; in addition, data will be used to evaluate the efficacy of both imaging systems and tracers in intraoperative detection and visualization of the tumor.

## 3 INNOVATIVE TRACERS CAPABLE OF CROSSING THE BBB

In addition to tracers that have already found clinical use, many others are the subject of intensive study and development. In this context, innovative approaches can be highlighted, which aim to exploit the whole set of molecular and physical–chemical characteristics characterizing the tumor microenvironment. In a completely analogous way to the development of new therapeutic agents, this line of research aims to obtain increasingly precise, safe, and economically viable tracers. In addition, the possibility of designing new (chemical) tracers that combine different modes of action, as well as being able to flexibly modulate their use (*e.g*., on‐site activation), is of great interest.

### 3.1 Peptide and protein‐based compounds

Peptide and protein‐based delivery systems have shown considerable potential in delivering imaging across the BBB, holding promise for targeted and efficient treatment of brain tumors. The synthetic versatility of peptides, in particular, has made them extensively used delivery agents, either directly conjugated to drug molecules or used to functionalize drug‐loaded nanocarriers, including liposomes and synthetic or biogenic NPs. In addition, as explained in the previous section, peptides and proteins are increasingly being used as imaging probes to visualize tumors, guide their resection, or monitor their response to therapeutic treatment.^59^ A dual mode of action is possible for peptides and proteins as delivery tools, either as BBB crossing agents or active targeting molecules through binding to tumor overexpressed receptors. However, both EGFR antibody and CTX peptide are not the only molecules that have proven to be useful for tumor targeting. Indeed, numerous sequences are reported, and individual mechanisms are described. For the most comprehensive list currently available, refer to Parrasia et al.^60^

#### 3.1.1 BBB crossing peptides

Different mechanisms can account for these shuttling properties, including interaction with transporters, receptors, or direct translocation across the BBB, thanks to favorable physicochemical properties.^61^ Here, some of the most recurrent mechanisms are briefly outlined. The first approach relies on the use of peptides/proteins showing affinity for BBB endothelial cell receptors, facilitating their transport across the barrier through receptor‐mediated transcytosis. For instance, angiopep‐2 has the ability to bind the low‐density lipoprotein receptor‐related protein 1, which is highly expressed in BBB endothelial cells. A similar mechanism applies to the ApoE peptide derived from the ApoE protein, which is involved in lipid metabolism and transport. These features were, for instance, used to create MRI/NIR fluorescence dual‐modal imaging nanoprobes, which pinpoint malignant gliomas and direct precise excision,^62^ or to deliver novel theranostics.^63^, ^64^ Other examples include the specific binding to the acetylcholine receptor, as in the case of RVG peptide, or the targeting of transferrin or insulin receptors.^65^

Alternative mechanisms to BBB crossing can see both peptide‐mediated direct translocation, typical of cell‐penetrating peptides (CPPs), and transient disruption of the tight junctions between endothelial cells of the BBB. These BBB‐opening properties are shown in some peptide analogs of bradykinin, and can be exploited to improve the delivery of anticancer drugs to the brain.^66^, ^67^ Moreover, in general, the direct translocation of CPPs is typically triggered by the interaction between positively charged CPP residues and negatively charged membrane elements, causing either permanent or temporary membrane instability induced by the folding of the lipid membrane. Several mechanistic models for direct penetration have been proposed, as extensively reviewed elsewhere.^68^ The transactivating transcriptional activator peptide is likely the most popular and broadly used CPPs, which was shown to efficiently cross the BBB and enhance the delivery of therapeutic agents, including anticancer drugs, to the brain.^60^ Accordingly, CPPs were reported as effective tools to translocate fluorescent probes and sensors across the BBB^69^, ^70^ to monitor the tumor state and anticancer drug pharmacodynamics. In addition to peptides, proteins themselves can be fluorescently labeled and serve as fluorescent tracers of damaged BBB.^71^, ^72^

#### 3.1.2 Tumor‐targeting peptides

Tumor‐targeting peptides are short sequences of amino acids that possess a specific affinity for molecules or receptors overexpressed on the surface of tumor cells or other elements of the tumor microenvironment (e.g., α_ν_β_3_), including tumor vessels. These peptides can be utilized for targeted drug delivery, imaging, and diagnosis of various types of tumors, including brain tumors.^73^, ^74^ For example, peptides containing Asn‐Gly‐Arg (NGR), Arg‐Gly‐Asp (RGD), or *iso*Asp‐Gly‐Arg (*iso*DGR) motifs selective for CD13 or integrins overexpressed by tumor vasculature have been explored in this sense. Many other receptors are overexpressed on the surface of brain tumor cells and have been exploited accordingly. Examples include peptides targeting EGFR (see Section 2.2), platelet‐derived growth factor receptors, vascular endothelial growth factor receptors, and insulin‐like growth factor receptors.^75^ Also, luteinizing hormone‐releasing hormone (LHRH) receptors are overexpressed in certain types of brain tumors, such as gliomas, as well as somatostatin receptors (SSTRs), particularly in the case of neuroendocrine tumors. Peptide compounds such as triptorelin and goserelin (LHRH), and octreotide and lanreotide (SSTRs), have been consequently investigated for their tumor‐targeting potential.^76^, ^77^, ^78^

A radically different and alternative approach to tumor targeting is well exemplified by pH‐insertion peptides (pHLIPs), a class of molecules that possess the ability to undergo structural changes in response to pH variations.^79^, ^80^ In particular, these peptides can sense the acidic microenvironment surrounding tumor cells, a well‐known hallmark of cancer, which is due to the increased use of glycolysis by cancer cells, and by the abundance of carbonic anhydrase proteins on the cancer cell surfaces. The key characteristic of pHLIP peptides is their pH sensitivity. These peptides indeed undergo conformational changes in response to the acidic pH found in tumor tissues. In particular, the pH from physiological conditions to the surroundings of tumor cells induces the loss of peptide‐charged states and an increase in overall hydrophobicity, which then drives pHLIPs partitioning across the membrane bilayer. This process can enable the controlled release of therapeutic agents at the tumor site, by exploiting the acidic pH gradient between tumor tissues and healthy tissues. Besides being widely investigated for therapeutics delivery, including small‐molecule drugs and nucleic acids,^81^, ^82^, ^83^ this approach was also successfully applied for imaging agents.^79^, ^80^ Promising results were obtained in FGS using pHLIP technology, facilitating the visualization of cancerous lesions, including primary tumors and submillimeter‐sized metastatic lesions, as recently demonstrated in an ongoing clinical trial for breast cancer imaging (ClinicalTrials.gov Identifier: NCT05130801).

Overall, the selection of the most suitable and effective systems for tumor targeting may vary depending on the specific type and characteristics of brain tumors.

Peptide‐based delivery systems have shown considerable potential in delivering anticancer therapeutics across the BBB, holding promise for targeted and efficient treatment of brain tumors. The synthetic versatility of peptides, in particular, has made them extensively used delivery agents, either directly conjugated to drug molecules or used to functionalize drug‐loaded.

### 3.2 APC analogs

**FIGURE 3:**
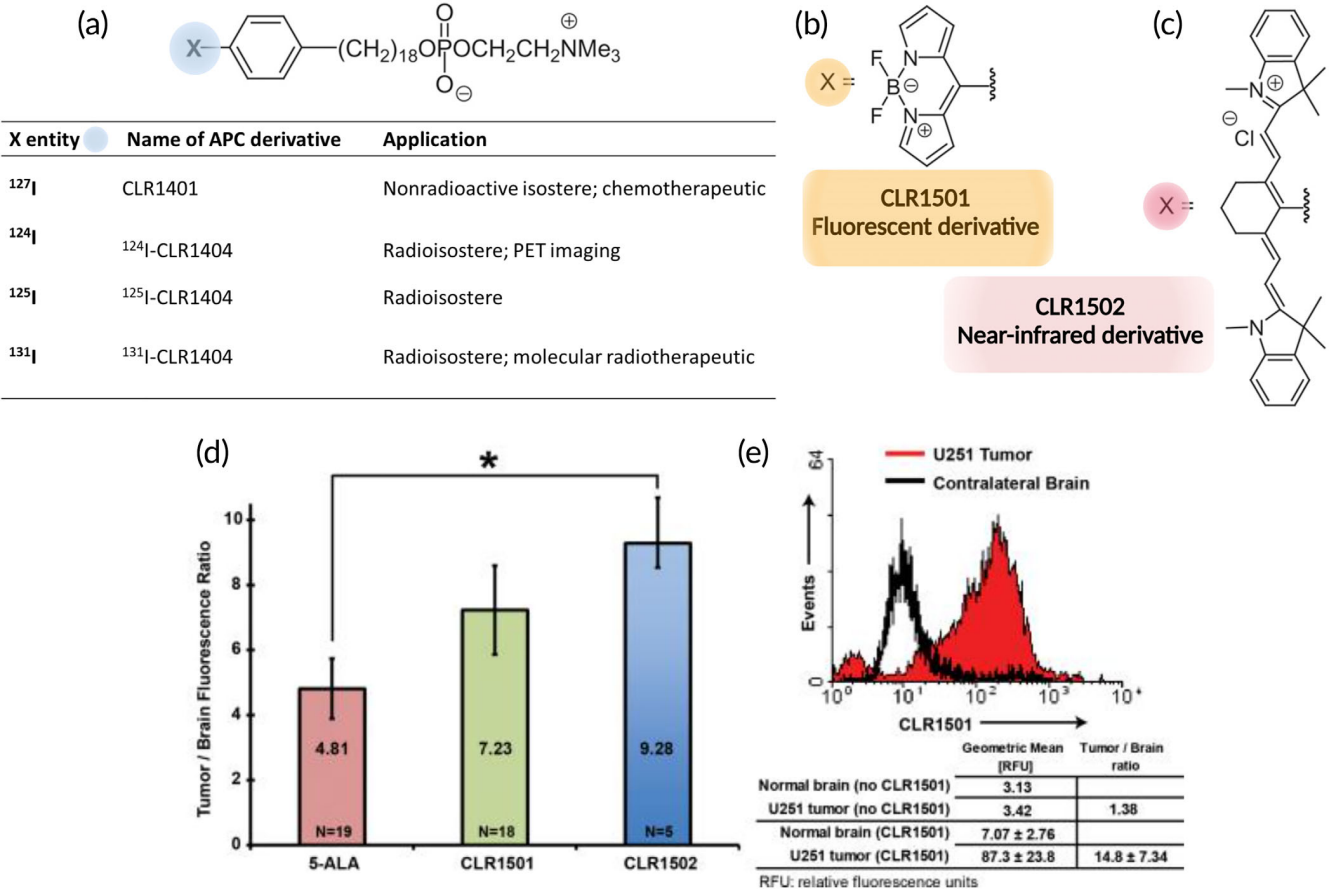
(a–c) Chemical structures of specific APC derivatives. In detail, CLR1404 is developed as a radioactive (a), fluorescent (b), or near‐infrared compound (c). (d, e) CLR1501 performance in U251‐derived orthotopic xenograft. (d) CLR1501‐related tumor to brain fluorescence ratio compared with 5‐ALA and near‐infrared CLR1502; (e) flow cytometry of CLR1501+ cells versus normal brain. Reproduced with permission from Swanson and colleagues.^28^, ^84^

Another promising strategy for targeting cancer tissues is using APC analogs functionalized with imaging moieties. In fact, it has been shown that solid tumors are richer in phospholipid ethers than normal cells. From this evidence, iodinated APC derivatives, functionalized with 124I and 131I, have been developed as “diapeutic” agents working as PET tracers and radiotherapeutics, respectively.^84^ Among APC derivatives, those bearing alkyl chains with a number of –CH_2_ groups higher than 11 demonstrated the highest tumor selectivity.^85^ The preferential uptake in cancer cells with respect to normal cells seemed to be related to the presence of richer areas of lipid rafts (enriched in cholesterol) in the former cells, which work as tumor gates promoting both uptake and retention of these agents in the plasma membranes^86^ of cancer cells. The major strength of these compounds is their broad‐spectrum application in different types of cancers and co‐localization with lipid membranes. Both human glioblastoma and glioblastoma stem‐like cells (GSC) were successfully labeled with the iodinated octadecyl phosphocholine derivatives (CLR1404) (see Figure 3a–c), and their ability to target glioblastoma malignancies was also tested in GSC‐derived orthotopic murine xenografts, with evidence that benign and premalignant tumors were scarcely labeled.^84^ The same authors administrated ^124^I‐CLR1404 to a small number of lung and brain cancer patients. PET analysis revealed both tumor uptake and retention, demonstrating their possible use for postoperative treatment to visualize any remaining cancer cells after tumor removal. This approach was also applied for FGS glioma resection in murine glioblastoma models. In particular, two different derivatives were synthesized, CLR1501 and CLR1502, functionalized with a green dye and an infrared dye, respectively. The results were reported in comparison with the standard tracer for FGS, 5‐ALA.^28^ Data showed that while CLR1501 had comparable results to 5‐ALA, the IR emitting compound (CLR1502) demonstrated a higher tumor to normal brain fluorescence ratio. This is likely due to better tissue penetration and a lower tissue autofluorescence background (Figure 3d,e). This approach is promising, and many derivatives have been investigated in clinical trials and their clinical translation is related to their PK profiles, which resemble those of fatty acids (FA). In silico docking analysis coupled with in vitro/in vivo portioning experiments and in vivo PK experiments on these derivatives were performed, providing guidelines for an optimized molecular design of APC analogs.^87^ In fact, long plasma residence times after administration are suboptimal for imaging tracers as they can impede obtaining a sufficient contrast in the tumor. The PK profile of APC derivatives is strongly affected by their binding to albumin and lipoproteins, and this should be carefully studied as it influences clearance and bioavailability. In fact, increased binding to lipoproteins seems to enhance the clearance rate of these compounds.

### 3.3 Activable imaging probes

Another class of possible selective imaging probes includes the activable ones: imaging probes triggered by either disease‐relevant enzymes^88^ or endogenously produced molecules overexpressed in cancer cells.^89^ The possibility of having imaging probes with an “on–off” response to tumor microenvironment represents an effective tool to selectively illuminate/darken cancer tissues. In this regard, a fluorescent probe activated by γ‐glutamyltranspeptidase (GTT), overexpressed in glioma cells, was developed. In particular, this reporter is composed of a Bodipy scaffold (fluorescent moiety) and a cys‐γ‐glu sequence sensitive to GTT. GTT promotes a selective cleavage of the peptide moiety, abruptly changing the photophysical features of the reporter and enabling a live‐time detection of the enzyme activity. The reporter was topically sprayed on U87 tumor xenograft‐bearing mice and also applied ex vivo on human tissue specimens of glioma (Grade II–IV) from patients, demonstrating to be very sensitive and raising a strong difference in signal between healthy and cancer tissues.^90^ The same approach has been shown to be also effective in determining glioma recurrence. GTT expression was correlated with an increase in malignancy of statistical numbers of glioma samples.^91^

The cancer microenvironment is a complex matrix continuously investigated by the research community; it has been shown that many cancer cells, for example, have a high expression of hydrogen sulfide (H_2_S) producing enzymes and the increased production of H_2_S affects cancer pathophysiology.^89^ For example, it activates tumor angiogenesis and cell proliferation.^92^ Thus, imaging probes (fluorescent and luminescent) for H_2_S detection in cancer cells were developed.^89^, ^93^ Also, in this case, the reporters are designed to undergo a chemical reaction triggered by the targeting molecule (H_2_S), strongly altering their photochemical behavior, enhancing (or quenching) fluorescence emission at a specific wavelength.^94^ This mechanism should allow to selectively distinguish the cancer cells from healthy cells, in which the triggering molecule should be either less expressed or absent.

## 4 NANOSYSTEMS FOR FGS

**FIGURE 4:**
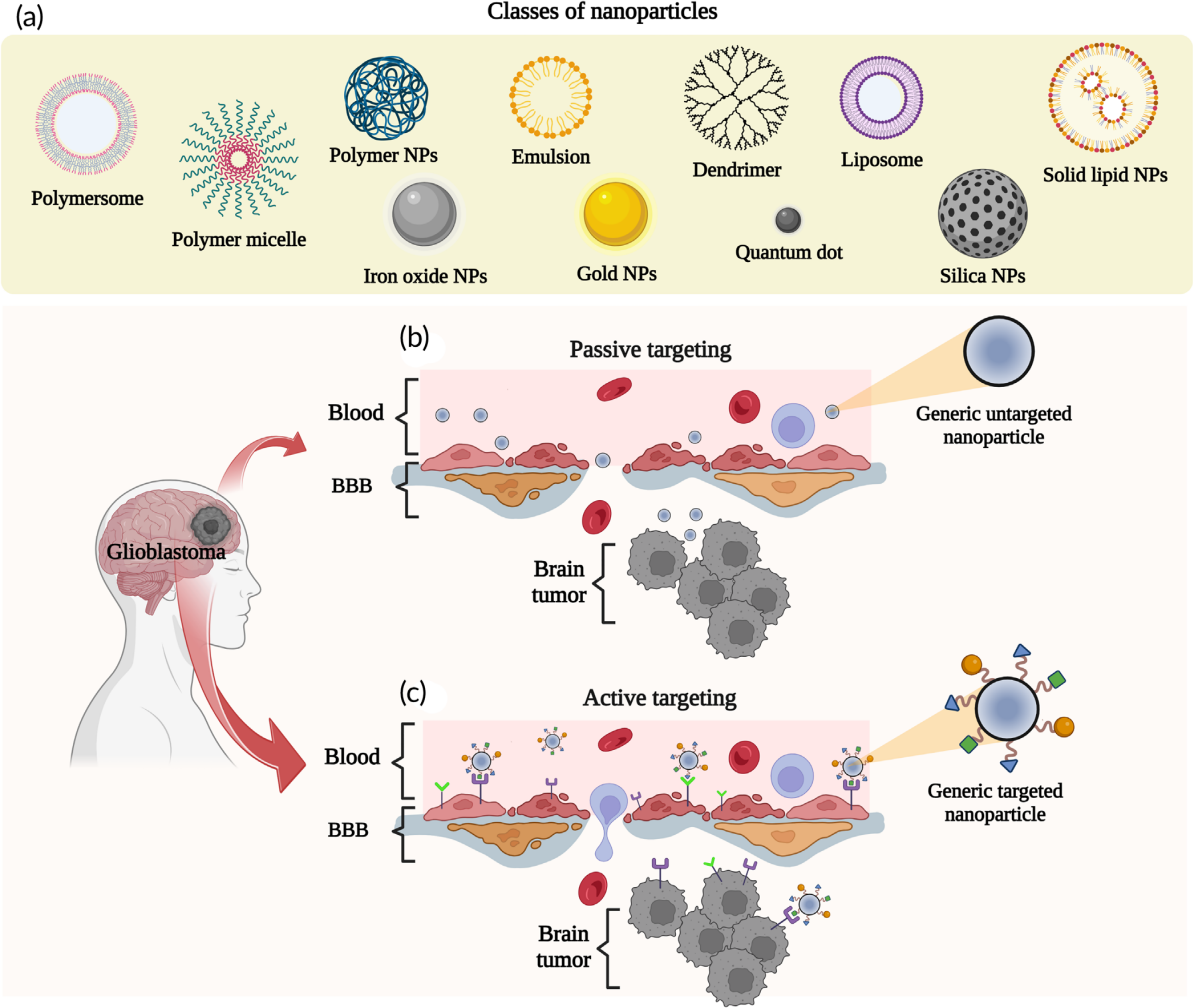
(a) Schematic representation of different types of nanoparticles (NPs) used for cancer targeting; transport mechanisms of generic NPs across the blood–brain barrier (BBB). (b) Passive targeting includes paracellular and transcellular diffusion of small (≤10 nm) and highly lipophilic NPs. (c) NP‐mediated targeted delivery to glioblastoma cells. This active mechanism exploits the conjugation of targeting moieties to cargo‐carrying NPs. The diffusion of targeted NPs across the BBB is ensured by carrier‐mediated transcytosis, adsorptive‐mediated endocytosis, or receptor‐mediated endocytosis Created with BioRender.com.

In addition to several “free” imaging agents capable of successfully highlighting resection margins, we will now focus on nanoscaled systems (Figure 4). They gained significant attention in the last decades, not only for their crucial therapeutic potential^95^ but also for their strategic capacities to monitor glioblastoma in real time. Specifically, the use of nanoscaled systems might improve glioblastoma labeling effectiveness and safety by protecting imaging tracers from enzymatic degradation, extending plasma half‐life, and increasing tumor accumulation.

**FIGURE 5:**
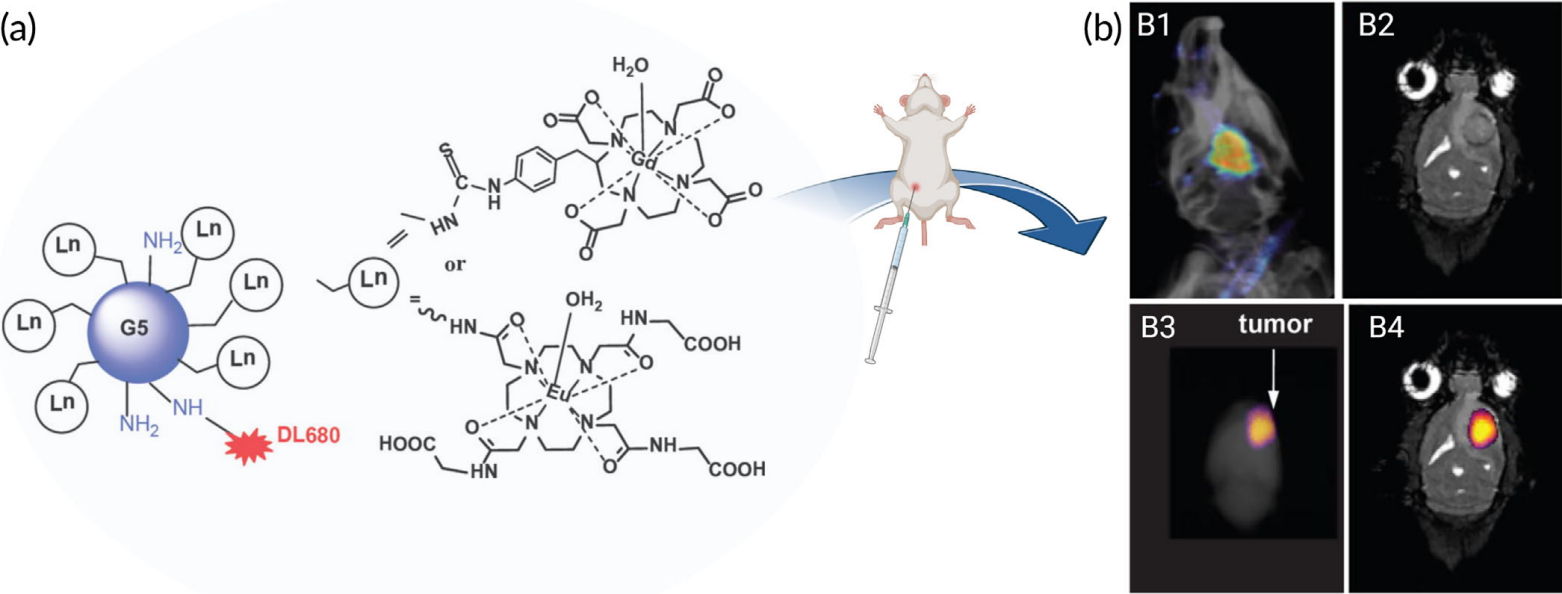
(a) Schematic representation of Gd‐based (DOTA) or Eu‐DOTA‐Gly4 agent conjugated on the surface of a G5 PAMAM dendrimer with DyLight (DL680). (b) Nanoparticles (NPs) visualization after administration in a U251 glioma rat model. (b1) An in vivo fluorescent image of the rat head overlayed on an x‐ray image confirms the presence of Eu‐DOTA‐Gly4‐G5‐DL680 NPs in the brain tumor. (b2) Coronal MRI image showing the location of the tumor. (b3) Overlay of the ex vivo fluorescent image on x‐ray acquisition of the whole brain. (b4) Overlay of the ex vivo fluorescent image on the brain MRI acquisition. Reproduced with permission from Gonawala et al.^104^

Undoubtedly, a key role is played by the NPs features, especially regarding their physicochemical properties such as size, shape, surface charge, and composition^96^ (Figure 4a). The primary mechanism for NPs delivery is certainly through the leaky blood vessels and compromised lymphatic system of the tumor, known as passive targeting (Figure 4b). Specific features such as small sizes, stealth surface coatings (typically poly(ethylene glycol) [PEG]), and negative/neutral surface charges enhance passive and nonspecific tumor uptake of NPs. In this way, serum protein binding could be prevented, ensuring longer plasma half‐lives and thus causing consequent accumulation in cancer tissues.^97^, ^98^ However, although a higher BBB permeability related to this pathological condition^99^ might render the NPs passage easier, it is often not enough to guarantee their relevant penetrance. This is a consequence of the highly heterogeneous and less EPR effect in glioma in comparison with other cancer subtypes.^100^ Indeed, BBB seems to be still intact in some of the tumor regions, compromising the passive tumor targeting efficiency.^100^ Moreover, even if the EPR effect still remains a central paradigm in cancer nanomedicine, there are recent evidences suggesting that endothelial permeability is not the only mechanism responsible for the transport of NPs into solid tumors.^101^ For all these reasons, there is a continuing need to study the entry mechanisms of NPs within solid tumor and develop NPs capable of crossing the BBB and increase the intracranial availability of these imaging probes. In this context, Singh et al. proved that the simultaneous encapsulation of curcumin and photonic CbV dyes into NPs (TPNs) could easily cross the BBB and provide not only a therapeutic agent, but also an optical tracer for in vivo imaging of glioblastoma.^32^ This was possible by designing ultrasmall NPs obtained by self‐assembly of a triblock polymeric amphiphile with known penetrating properties.^102^, ^103^ Another nanoscaled platform showing great potential for FGS in glioblastoma is represented by dendrimers. Specifically, a bimodal MRI and NIR fluorescence imaging agent was synthesized by conjugating a Gd‐based agent and a fluorescent dye (DyeLight680) to PAMAM dendrimers^29^ (Figure 5a). Importantly, the proposed systems showed a glioma‐specific accumulation after their systemic administration in a rat model^104^, ^105^ (Figure 5b).

The development of nanoscaled systems often offers a way to improve tumor selectivity of freely circulating small‐molecule compounds, which normally could also diffuse into healthy tissues, complicating their ability to define tumor margins. An example of this is the passive fluorescent agent Evans blue (EB), which showed an improved sensitivity of 89% in discriminating brain tumor, when encapsulated in liposomal NPs. However, despite these, EB‐loaded liposomes do not stain healthy brain tissue, in vivo results in rat models proved an underestimation of the cancer margins on the order up to hundreds of micrometers.^106^ This inaccuracy represents an important hurdle for a clinical translation. In this context, several innovative strategies have been developed to enhance liposomes' ability to reach the brain and target the tumor. One promising approach involves liposome decoration with tumor‐specific pH‐responsive peptides. Indeed, the resulting pH‐sensitive liposomes can be identified as carriers capable of selectively responding to the acidic pH environment in gliomas, triggering the encapsulated probe release. The idea is to exploit the specific glioblastoma acidic pH environment^107^, ^108^ to improve glioma targeting. This liposome‐based strategy has been mainly investigated for the encapsulation of anticancer drugs,^109^, ^110^ suggesting a significantly increased accumulation at slightly acidic pH (pH 6.8) compared with that at physiological value for both in vitro and in vivo applications.^109^ The evidence of a higher tumor selectivity of these modified liposomes suggests that this approach can also be used to deliver hydrophilic and hydrophobic fluorescent agents (i.e., *fluorescein* and *ICG*). Of note, as this approach is highly innovative, there are still no studies regarding it, and research in this field is growing. Interestingly, the use of pHLIPs represents a highly versatile attitude to enhance tumor targeting. Indeed, this approach has been extended to different nanomaterials (polymer, metal‐based NPs).^111^, ^112^

In summary, except for pH‐sensitive probes, many of the nanosystems described above show a limited sensitivity for tumoral cells. Indeed, they can nonspecifically diffuse through the brain, at times compromising healthy tissues.^113^ It is also for this reason that the delivery strategy known as active targeting is often preferred (Figure 4c). Nanosystem surfaces are usually modified with specific ligands that are selectively recognized by receptors overexpressed in glioblastoma cells, as previously discussed in Section 3.1.2.

**FIGURE 6:**
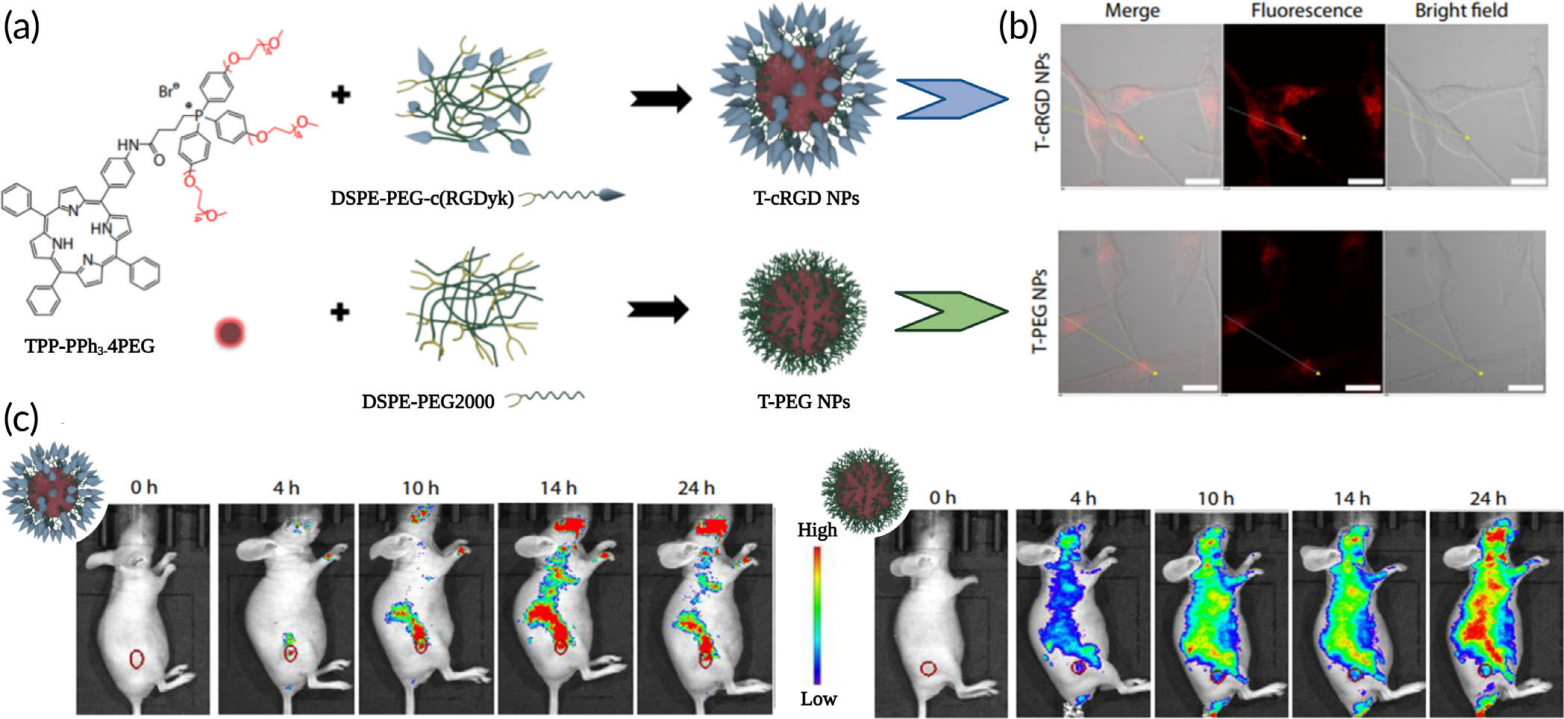
(a) Preparation of T‐cRGD NPs and untargeted T‐PEG NPs. (b) Fluorescence images of glioblastoma U87MG cells after incubation with T‐cRGD NPs or T‐PEG NPs for 16 h. (c) Fluorescence in vivo imaging of nude mice at different times after intravenous injection of T‐cRGD NPs (left) or T‐PEG NPs (right) (red circle: tumor position). Adapted and reproduced with permission from Jiang et al.^114^

In this context, RGD peptide‐decorated nanocarriers represent a successful approach. Several efforts have been made using cyclic RGD (cRGD) peptides, thanks to their stronger stability, selectivity, and binding affinity. In detail, Jiang et al. proposed a cRGD‐decorated porphyrin‐based sonosensitizer with a water‐soluble biological carrier polymer (T‐cRGD NPs) (Figure 6a,b).^114^ This strategy shows good translational chances considering that T‐cRGD NPs can easily accumulate into tumor tissues for in vivo fluorescence imaging and prove great therapeutic effects with good biocompatibility (Figure 6c).

**FIGURE 7:**
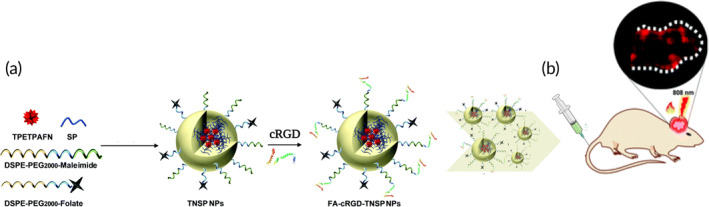
(a) Schematic illustration of FA‐cRGD‐TNSP nanoparticles (NPs) preparation and (b) in vivo glioblastoma targeting after intravenous NPs administration in tumor‐bearing mice. Adapted and reproduced with permission from Cai et al.^115^

In addition, cRGD can be combined with other glioblastoma‐targeting ligands (i.e., folate, FA) to increase labeling selectivity of tumor cells. This is the case of the organic NPs, namely FA‐cRGD‐TNSP, that have been developed by co‐encapsulating a fluorogen (TPETPAFN) with aggregation‐induced emission^115^ (Figure 7a). Of note, the proposed NPs show an efficient labeling of both the glioblastoma margin and inner tumor area after in vivo administration (Figure 7b). Nevertheless, recent studies showed the specific relevance of other cRGD‐decorated nano‐based technologies (quantum dots [QDs]^116^ and dendrimers^30^) capable of efficiently imaging glioblastoma tumor margins. Importantly, the great potential of QDs for the detection and treatment of glioblastoma has been widely discussed also by Kantelhardt et al., where these nanosystems were coupled with monoclonal antibodies against EGFR, highly upregulated in many gliomas.^117^

However, the pressing need for improved visualization of glioblastoma margins and localized treatment focused the researchers' attention on the development of alternative strategies based on other targeting ligands, such as angiopeptide‐2 and transferrin. Although recent studies widely proved the great potential of angiopep‐2 to enhance the accumulation of desired therapies in gliomas,^118^, ^119^, ^120^ its use as a supporting tool for FGS is still poorly investigated. Indeed, the proposed fluorescent nanoplatforms are mainly combined with photo‐induced therapies (photothermal or photodynamic, PTT and PDT, respectively) as alternative promising modalities for glioblastoma treatment. In this context, Tsai et al. proposed a promising technology advancement based on the development of lanthanide‐doped upconversion NPs.^95^ These PEG/angiopep‐2 conjugated NPs ensured a glioblastoma‐specific delivery of photosensitizers (IR‐780 and temoporfin mTHPC) for an initial and efficient tumor imaging, followed by the externally triggered targeted therapy.

Lactoferrin (Lf) has been largely investigated as a possible target for glioblastoma imaging and therapy. In detail, Lf is a globular glycoprotein, which specifically binds to its receptor (LfR) present on BBB‐associated cells.^121^ Considering that LfR is expressed on cerebral microvascular endothelial cells in glioblastoma,^122^, ^123^ conjugated Lf nanocarriers can easily target glioma cells through LfR‐mediated endocytosis.^124^ Molecular targeting with Lf has been mainly performed with polymer‐based NPs as nanogels conjugated with Cy5.5‐tagged Lf, suggesting a significantly improved labeling of glioma cells.^125^ In the alternative, Zhou et al. proposed iron oxide NPs (SPIONs) loaded micelles, which were modified with both the Cy5.5 NIR fluorescent probe and Lf.^31^ These fluorescent/MRI bimodal micelles not only specifically targeted the glioma, but also provided support for surgical glioma resection in a mouse model.

As explained before for several peptides and proteins (see Section 3.1), these ligands are often exploited to cross the BBB through receptor‐mediated transcytosis. This is true not only for the angiopep‐2 and Lf proteins above described, but a similar approach applies to ApoE. Indeed, researchers recently discussed the potential of ApoE‐enriched protein corona around polymeric NPs as a targeting strategy for brain delivery.^126^ In addition, this protein can be directly linked to the NP surface^64^, ^127^ (such as polymersomes, ApoE‐PS). Because ApoE‐functionalized NPs are providing novel insights into glioma therapy through the design of targeted and efficient drug delivery systems, we believe that the same mechanism can be exploited to transport fluorescent tracers for image‐guided glioblastoma surgery.

**FIGURE 8:**
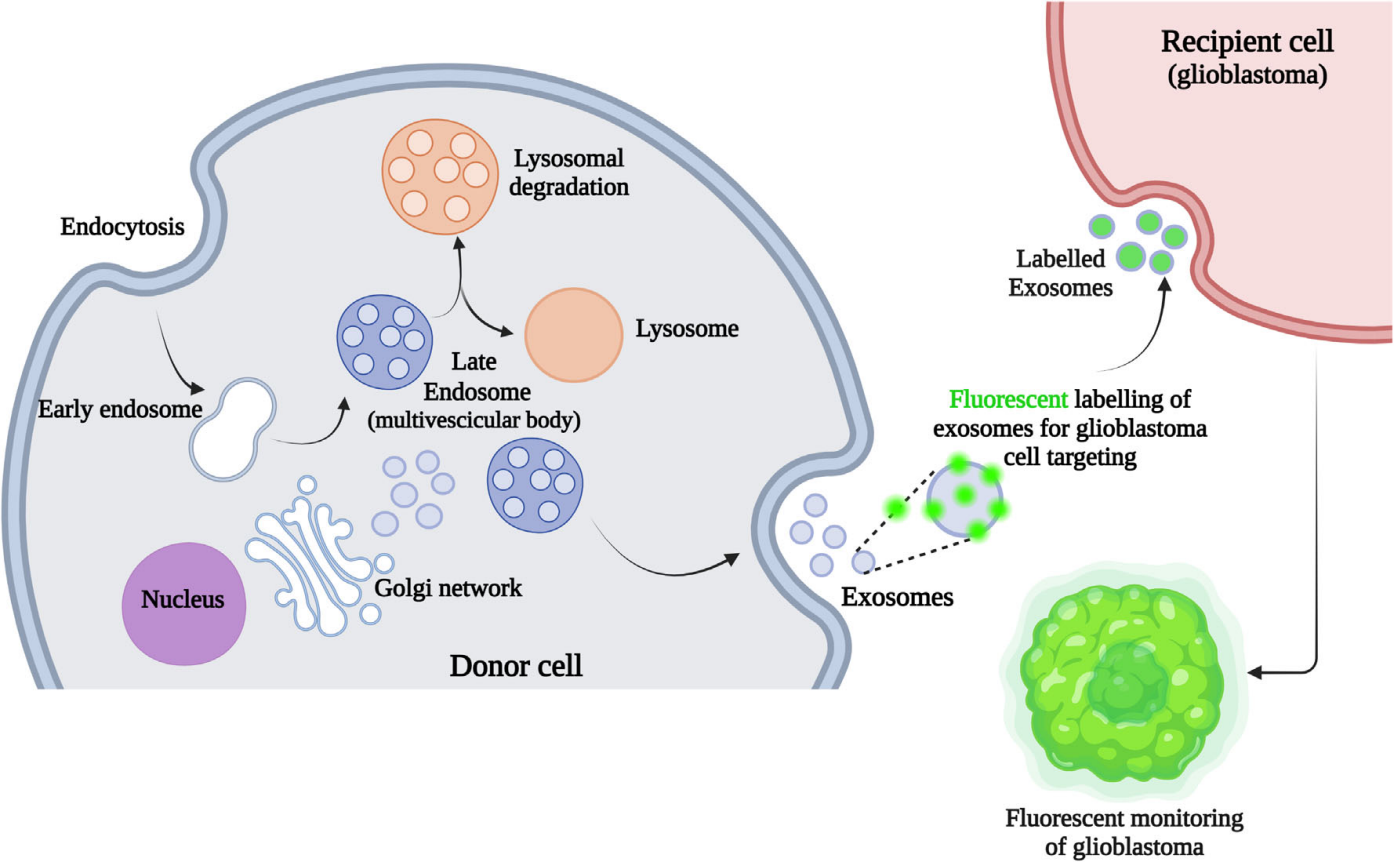
Schematic representation of the pathway involved in biogenesis of extracellular vesicles (EVs). In detail, formation of EVs starts with membrane endocytosis, allowing the generation of early endosomes that then mature into late endosomes or multivesicular bodies (MVBs). MVBs are carried to the plasma membrane through the cytoskeletal network, and exocytosis can occur, causing the release of EVs from the donor cell. The resulting EVs can be isolated and then fluorescently labeled (green spot), acting as a vehicle capable of selectively imaging recipient glioblastoma cells. Created with BioRender.com.

A radically alternative and innovative approach for image‐guided glioblastoma targeting is represented by extracellular vesicles (EVs) through the specific loading of fluorescent probes and the modification of exosomal membranes for imaging^128^ (Figure 8). Because EVs play key roles on the initiation, progression, and diagnosis of glioblastoma,^129^, ^130^, ^131^ as well as on the intercellular communication within the tumor microenvironment,^132^ they show great potential as delivery vehicles for cancer imaging and also therapy. Importantly, in addition to their abundance in body fluids, high stability, and low immunogenicity, they show a great ability to cross physiological barriers, including the BBB.^133^

In this respect, while many efforts have been addressed to the EV‐guided delivery of therapies for gliomas,^134^, ^135^, ^136^ the research focused on EVs as a supporting tool for image‐guided surgery is still growing.^137^ Furthermore, although recent advances have been made in the development of methods for the large‐scale production of EVs,^138^ the main weakness in the translation of this approach into clinical practice is the lack of a clinically relevant number of EVs, ensuring their integrity and biological activity.

## 5 CHARACTERISTICS OF IDEAL NEXT‐GENERATION AGENTS

As explained above, the established fluorescent agents used in surgical practice to improve intraoperative tumor identification and resection, although associated with a positive effect on EOR, present some limitations, such as poor selectivity, photostability, photosensitization, and high costs.

Therefore, there is a need to develop next‐generation agents with the capabilities to overcome the limitations related to the actual fluorescent approach to surgical resection of glioblastomas.

Next‐generation agents should be designed carefully considering the clinical/surgical needs together with the important biological barriers represented by the complex glioblastoma microenvironment. In fact, the ideal fluorescent probe should be designed to ensure specific and desirable properties, such as high binding affinity for tumor cells and excellent optical qualities, thus providing a high tumor/background ratio for improved contrast between tumor and normal tissues. A fine selectivity for glioblastoma cells can only be reached when the agents easily pass the altered BBB to localize into the brain area, where the tumor is located. For this purpose, some good candidates could be molecules with intrinsic shuttling properties (e.g., peptides and protein‐based compounds). Of note, active targeting is often preferred to increase the tumor selectivity and preserve healthy tissues. Indeed, many efforts aimed at the development of fluorescent biomarkers capable of recognizing molecules/receptors overexpressed on the surface of tumor cells. Another critical point for the proposed agent is its plasma stability. Specifically, the in vivo stability of the imaging tracer should be improved by limiting enzymatic degradation and thus increasing plasma half‐life. In this respect, the use of NPs could definitely help. However, specific strategies are required to either prevent the formation of a rich protein corona or control its composition on the NP surface, which can render tumor targeting inefficient. Finally, methods for large‐scale production of these agents should also be developed to ensure a reliable translation into clinical practice.

From a clinical/surgical point of view, there are different characteristics to be considered. The fluorescent agents should be easily intravenously administered, possibly close to the surgical procedure, to simplify the surgical workflow. This could also enable the possible utilization in urgent settings. They should have a favorable pharmacodynamic profile and low side effects including allergic reactions. In addition to the ability to easily pass the BBB, the ideal agent should selectively target the glioblastoma cells, independently of their biomolecular profiles, both at the central tumor core and at its margins of infiltration. Finally, the fluorescent properties of the molecule should allow for effective visualization of the tumor tissue by dedicated filters integrated into the three‐dimensional visualization systems (i.e., microscopes or exoscopes) in the operating room. This good visualization profile should also be demonstrated to provide an improvement in EOR and survival of glioblastoma patients.

## 6 CONCLUSIONS

Glioblastoma is one of the most aggressive brain cancer forms that can be diagnosed. Among all the currently available treatments, surgical resection represents the first crucial step capable of reducing the risk of tumor recurrence. In this context, FGS is increasingly used to improve the visualization of tumor margins, and thus guarantee the best surgical resection outcome. However, current fluorescent agents do not always succeed in the discrimination of tumor from normal parenchyma; thus, the surgical resection can be compromised. Here, we discuss the state of the art and propose possible solution in refining innovative fluorescent tools capable of more selectively targeting tumor during glioblastoma resection. The available agents would contribute to the development of more effective next‐generation molecules, which could in turn reduce the residual tumor in glioblastoma patients, and thus promote a decrease in tumor recurrence and patient mortality.

## AUTHOR CONTRIBUTIONS

**Cristina Chirizzi:** Conceptualization (equal); data curation (equal); formal analysis (equal); funding acquisition (equal); investigation (equal); methodology (equal); project administration (equal); resources (equal); software (equal); supervision (equal); validation (equal); visualization (equal); writing – original draft (equal); writing – review and editing (equal). **Serena Pellegatta:** Conceptualization (equal); data curation (equal); formal analysis (equal); funding acquisition (equal); investigation (equal); methodology (equal); project administration (equal); resources (equal); software (equal); supervision (equal); validation (equal); visualization (equal); writing – original draft (equal); writing – review and editing (equal). **Alessandro Gori:** Conceptualization (equal); data curation (equal); formal analysis (equal); funding acquisition (equal); investigation (equal); methodology (equal); project administration (equal); resources (equal); software (equal); supervision (equal); validation (equal); visualization (equal); writing – original draft (equal); writing – review and editing (equal). **Jacopo Falco:** Conceptualization (equal); data curation (equal); formal analysis (equal); funding acquisition (equal); investigation (equal); methodology (equal); project administration (equal); resources (equal); software (equal); supervision (equal); validation (equal); visualization (equal); writing – original draft (equal); writing – review and editing (equal). **Emanuele Rubiu:** Conceptualization (equal); data curation (equal); formal analysis (equal); funding acquisition (equal); investigation (equal); methodology (equal); project administration (equal); resources (equal); software (equal); supervision (equal); validation (equal); visualization (equal); writing – original draft (equal); writing – review and editing (equal). **Francesco Acerbi:** Conceptualization (equal); data curation (equal); formal analysis (equal); funding acquisition (equal); investigation (equal); methodology (equal); project administration (equal); resources (equal); software (equal); supervision (equal); validation (equal); visualization (equal); writing – original draft (equal); writing – review and editing (equal). **Francesca Baldelli Bombelli:** Conceptualization (equal); data curation (equal); formal analysis (equal); funding acquisition (equal); investigation (equal); methodology (equal); project administration (equal); resources (equal); software (equal); supervision (equal); validation (equal); visualization (equal); writing – original draft (equal); writing – review and editing (equal).

## CONFLICT OF INTEREST STATEMENT

Dr. Francesco Acerbi received honoraria from Carl Zeiss Meditec for lectures in International Meetings. Other authors declare no conflict of interest.

### PEER REVIEW

The peer review history for this article is available at https://www.webofscience.com/api/gateway/wos/peer-review/10.1002/btm2.10608.

## Data Availability

The authors confirm that the data supporting the findings of this study are available within the article.
